# Investigation of Variations in Brain Activity Associated with Dizziness and Head Position Alterations During Gait

**DOI:** 10.3390/healthcare14162490

**Published:** 2026-08-11

**Authors:** Sang Seok Yeo, Dong Hyun Byun, Fang He

**Affiliations:** 1 Department of Physical Therapy, College of Health Sciences, Dankook University, Cheonan-si 31116, Republic of Korea; yeopt@dankook.ac.kr; 2 Department of Special Creative Convergence, College of Rehabilitation Sciences, Daegu University, Gyeongsan-si 38453, Republic of Korea; bdh0529@hanmail.net; 3 Department of Public Health Science, Graduate School, Dankook University, Cheonan-si 31116, Republic of Korea

**Keywords:** dizziness, functional near-infrared spectroscopy, vestibular, visual, sensory conflict

## Abstract

**Highlights:**

**What are the main findings?**

- fNIRS revealed altered brain activation patterns in people with dizziness during gait.
- Head position changes showed different cortical responses between the dizziness and control groups.

**What are the implications of the main findings?**

- The altered cortical activation suggests changes in multisensory integration and sensory reweighting in individuals with dizziness during movement.

**Abstract:**

**Background/Objectives**: Dizziness is a common clinical symptom that can develop from various diseases and affect activities of daily living. This study investigated and compared brain activation in people with and without dizziness during different head position gait sessions using functional near-infrared spectroscopy (fNIRS). **Methods**: We included eight adults with symptoms of dizziness as the dizziness group and eight control participants without dizziness. Dizziness symptoms were evaluated using the Dizziness Handicap Inventory (DHI) and Vertigo Symptom Scale—short form (VSS-sf). **Results**: Compared with the control group, we found the activation of the bilateral middle temporal gyrus (MTG) when looking forward, the activation of the left superior parietal lobule (SPL) and right angular gyrus (AG) when looking upward, the activation of the left AG and the deactivation of the right MTG when looking rightward, the activation of the right SPL when looking leftward, and the activation of the left MTG and the deactivation of the right MTG when looking downward in the dizziness group at an exploratory, uncorrected threshold (*p* < 0.05). Notably, while all identified regions exhibited large effect sizes (Cohen’s d > 0.8), only the activation in the left SPL (channel 11) remained statistically significant after false discovery rate (FDR) correction when looking upward compared with the control group (corrected *p* < 0.05). **Conclusions**: These exploratory and hypothesis-generating findings suggest that altered cortical activation associated with multisensory integration may reflect changes in sensory reweighting during gait in individuals with dizziness under different head position conditions.

## 1. Introduction

Dizziness, defined as a feeling of spatial disorientation, imbalance, and incorrect perception of movement, can develop as a symptom of various diseases [1]. Dizziness can affect activities of daily living, such as balance and walking, and is a risk factor for falls in the elderly or clinical populations [2,3]. However, a clear diagnosis of dizziness is difficult because a variety of diseases can cause dizziness, and the diagnostic report and severity of dizziness usually depend on the surrounding environment, age, or physical condition [4].

Balance is a key factor in maintaining the center of mass and reducing the risk of falls, requiring complex integration of the visual, vestibular, and somatosensory systems [5]. In particular, loss of balance due to a decrease in vestibular function can interfere with activities of daily living and lead to secondary problems such as falls and fractures [6]. Previous research has shown that the decline or loss of vestibular function has a significant impact on dizziness because vestibular input plays an important role in spatial orientation [2,3]. Vestibular dysfunction (caused by the peripheral or central vestibular system) may manifest as dizziness, spatial disorientation, and imbalance [7].

Dizziness is related to injury of the parietal and temporal cortices, which involve the central areas for higher-order neural conversion and play an important role in multimodal integration by receiving signals from various senses related to the vestibular system [8,9]. According to some functional magnetic resonance imaging (fMRI) studies on cerebral activity in adults with right-dominant hands, there is a significant difference in cerebral activity between individuals with and without dizziness [10,11]. Previous studies have reported that different head postures result in different sensory signals from both the head and neck evoked by a perturbation and influence brain activity, even if the change in head posture does not alter the position of the body’s center of mass during a normal gait session [12,13]. However, studies investigating the activity characteristics of the cerebral cortex of the parietal and temporal lobes in individuals with dizziness symptoms according to their head posture during gait are lacking. Therefore, this study aimed to investigate and compare the cortical activity during normal gait with changes in head posture between people with and without dizziness using functional near-infrared spectroscopy (fNIRS).

## 2. Materials and Methods

### 2.1. Participants

Sixteen right-handed adults (14 females, 2 males; aged 20–35 years) were recruited for the study. After completing the Dizziness Handicap Inventory (DHI) and Vertigo Symptom Scale—short form (VSS-sf), all participants were divided into two groups: (1) a dizziness group including adults with symptoms of dizziness (8 females, DHI ≥ 16 and VSS-sf ≥ 12) and (2) a control group comprising adults without dizziness (6 females, 2 males, DHI < 16 and VSS-sf < 12). Based on self-reported medical history, participants in the dizziness group had no previous diagnosis of peripheral or central vestibular disorders. The inclusion criteria were as follows: (1) no history of musculoskeletal, neurological, or vestibular disorders; (2) no history of visual or cognitive impairment; (3) the ability to walk, maintain balance, and perform activities of daily living independently. The exclusion criteria included: (1) severe neck pain or limited cervical range of motion; (2) a self-reported history of vestibular disorders (e.g., Meniere’s disease, benign paroxysmal positional vertigo, or vestibular neuritis); and (3) known skin hypersensitivity or allergy that could interfere with the placement of the fNIRS optodes. All participants provided informed consent, and the study was approved by the Institutional Review Board of Dankook University, Republic of Korea (DKU 2023-06-017-003).

### 2.2. Measurements

#### 2.2.1. Functional Near-Infrared Spectroscopy (fNIRS)

**Figure 1:**
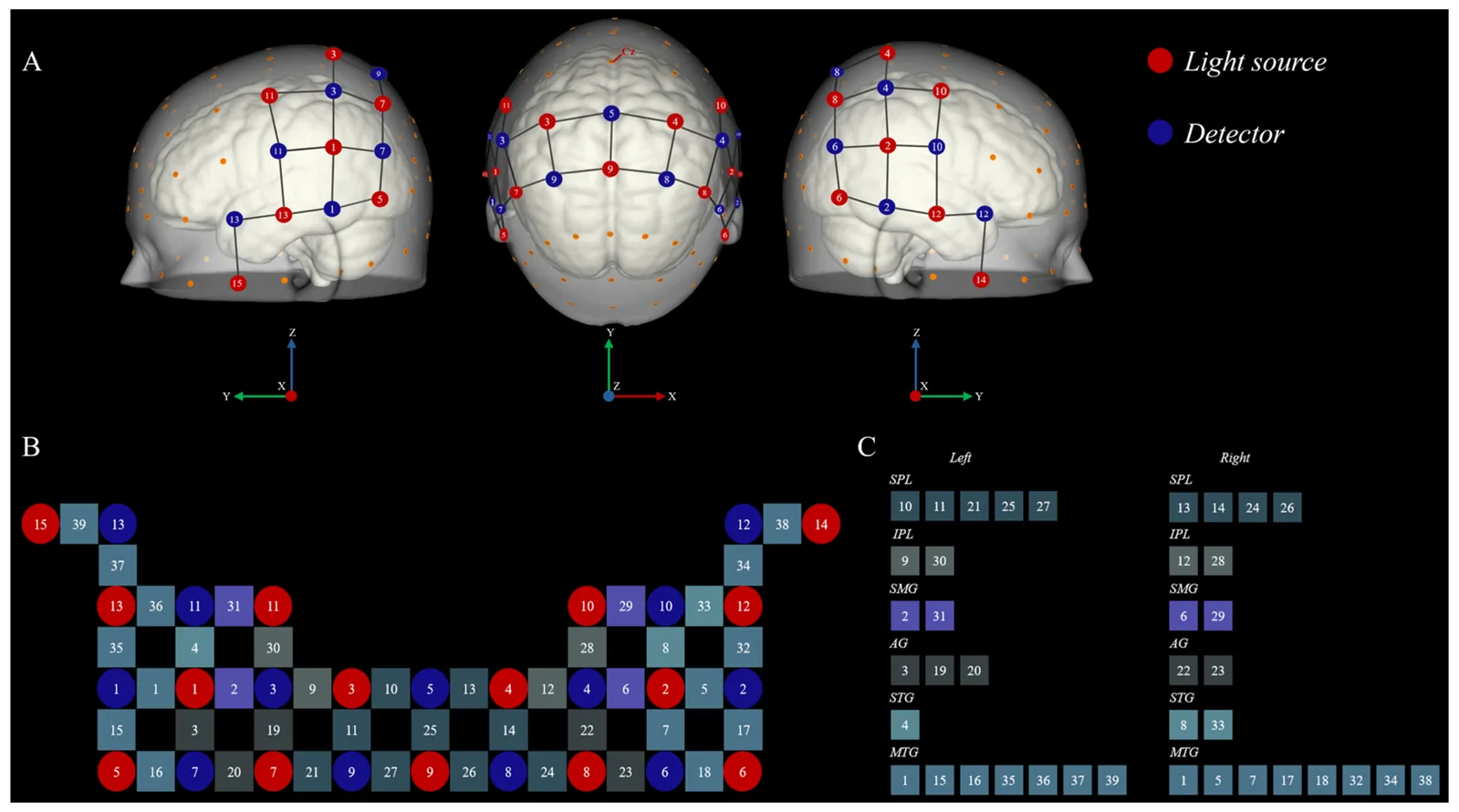
fNIRS optode placement and channel configuration. (**A**,**B**) fNIRS optode placement; the fifteen red and thirteen blue circles represent the positions of the light sources and detectors, respectively, with the digits inside indicating their corresponding indices. (**C**) Channel configuration and regions of interest (ROIs); the digits from 1 to 39 represent the channel numbers. SPL: superior parietal lobule, IPL: inferior parietal lobule, SMG: supramarginal gyrus, AG: angular gyrus, STG: superior temporal gyrus, MTG: middle temporal gyrus.

Continuous wave fNIRS (NIRSport 2, NIRx Medical Technologies LLC, Berlin, Germany) at a sampling rate of 10.8507 Hz was used to record hemodynamic responses during the gait sessions. NIRSport 2 is a wearable multi-channel fNIRS system designed to maximize the signal-to-noise ratio in free subjects because of its high-quality components, extensive versatility, and modularity [14]. This study included 15 sources and 13 detectors to record the optical light intensity at two different wavelengths (760 nm and 850 nm). The placement of the optodes was determined using the NIRsite 2021.4 software (NIRx Medical Technologies, LLC, Berlin, Germany) and the fNIRS Optode’s Location Decider toolbox (fOLD), using the Jülich histological atlas (SPM Anatomy Toolbox, Forschungszentrum Jülich, Jülich, Germany) for anatomical labeling [15,16,17]. FOLD toolbox was run in MATLAB 2023b (MathWorks, Inc., Natick, MA, USA) that computes the optimal optode placement in the international 10–20 system to cover specific brain areas. The distance between each source and detector was 3 cm, and 39 channels were used for data acquisition. This study set the regions of interest (ROIs) related to dizziness and vertigo as follows: bilateral superior parietal lobule (SPL), inferior parietal lobule (IPL), supramarginal gyrus (SMG), angular gyrus (AG), superior temporal gyrus (STG) and middle temporal gyrus (MTG) (Figure 1) [18,19].

The experiment utilized a block design paradigm consisting of alternating 15 s rest periods (standing still) and 15 s walking periods, repeated for three cycles per condition. During the walking periods, participants were instructed to walk in a straight line at their daily, self-selected comfortable pace. To investigate the effect of head posture, participants performed the gait sessions under five conditions in a fixed order: the head positioned forward, upward (neck extension), rightward (neck rotation), leftward (neck rotation), and downward (neck flexion). To ensure safety and accommodate individual anatomical variations, participants were instructed to move their heads to their maximum comfortable range for each target direction, with their gaze aligned with the head position, rather than strictly standardizing a fixed angle. Furthermore, a 10 min rest interval was provided between consecutive head position tasks to minimize potential carryover effects of dizziness, fatigue and habituation.

#### 2.2.2. Questionnaire

(1) Dizziness Handicap Inventory (DHI)

The DHI was established to assess the impact of dizziness and balance problems on the quality of daily life. This scale is scored on a scale ranging from a minimum of 0 to a maximum of 100; the questionnaire contains 25 items, with the answers graded as follows: yes, 4 points; sometimes, 2 points; and no 0 points. The questionnaire is divided into three subscales: physical (28 points), emotional (36 points), and functional (36 points) factors related to activities. The participants were evaluated based on the scores as follows: mild dizziness (16–34 points), moderate dizziness (36–52 points), severe dizziness (≥54 points) [20,21].

(2) Vertigo Symptom Scale—short form (VSS-sf)

The VSS-sf is a self-report instrument, comprising 15 questions, that covers the frequency and severity of dizziness symptoms over 1 month. Each item of the VSS-sf is scored on a 5-point scale (ranging from 0 to 4 points), with the results categorized as follows: 0 points (never), 1 point (a few times), 2 points (several times), 3 points (often), and 4 points (always). The total score ranges from 0 to 60 points. The higher the total score, the higher the frequency of symptoms. Dizziness was defined in participants with a score ≥12 points on the total scale [22,23].

### 2.3. Data Analysis

The fNIRS data were processed using the Near-Infrared Spectroscopy Laboratory (NIRSlab) version 2019.04 (NIRx Medical Technologies LLC, Berlin, Germany) by removing discontinuities and spike artifacts. Subsequently, the data quality was checked by setting the coefficient of variation (CV) to 15; the data were filtered in a band-pass of 0.001–0.20 Hz with a 15% roll of width to eliminate the effects of heartbeat, respiration, and low-frequency signal drifts for each wavelength [24]. Additionally, any changes in optical densities were converted using the modified Beer–Lambert law. The molar extinction coefficients and differential path length factors (DPF) of 7.25 (760 nm) and 6.38 (850 nm) were derived from the standard W.B. Gratzer spectrum. This study focused on HbO for further analysis, as it is more sensitive to locomotion-related tasks than HbR [25].

For topographic and statistical analyses of the fNIRS data, the Statistical Parametric Mapping NIRS-SPM (SPM 8) tool was used. The first-level (SPM-1, within-subject) analysis was performed to estimate task-related activation separately for each measurement channel. In SPM-1 analysis, no pre-whitening was used to analyze the data or consider the hemodynamic response function (HRF). In addition, this study applied the discrete cosine transformation (DCT) time parameter with a cutoff time of 128 s and the Gaussian full width at half maximum (FWHM) 4 model [26]. A general linear model (GLM) was constructed based on the HbO signals, while a design matrix was set up to contrast the remaining (0) and task (1) [27]. Based on the SPM-1, the second-level (SPM-2, between-subject) analysis was employed to perform group analysis and compare cortical activation between the experimental and control groups. Given the exploratory nature of this study and the relatively small sample size, between-group comparisons were initially evaluated using an uncorrected channel-level threshold (*p* < 0.05), followed by false discovery rate (FDR) correction to account for multiple comparisons. Both uncorrected and FDR-corrected results are reported where applicable. Effect sizes (Cohen’s d) were also calculated for each between-group comparison to quantify the magnitude of the observed differences.

### 2.4. Statistical Analysis

Spatiotemporal parameters were analyzed using SPSS software (version 25.0; SPSS, Chicago, IL, USA). The Shapiro–Wilk test was used to check the normality of HbO, while the χ^2^ test and independent *t*-test were used to identify significant differences in the demographic data between the two groups during gait sessions with changes in head posture.

## 3. Results

There were no significant differences in gender or age between the groups (*p* = 0.131 and *p* = 0.234, respectively). The independent *t*-test on the DHI and VSS-sf scores quantifying subjective feedback from the participants in the two groups showed statistically significant differences (*p* < 0.05) (Table 1).

### 3.1. Cortical Activations of HbO in the Dizziness Group

**Figure 2:**
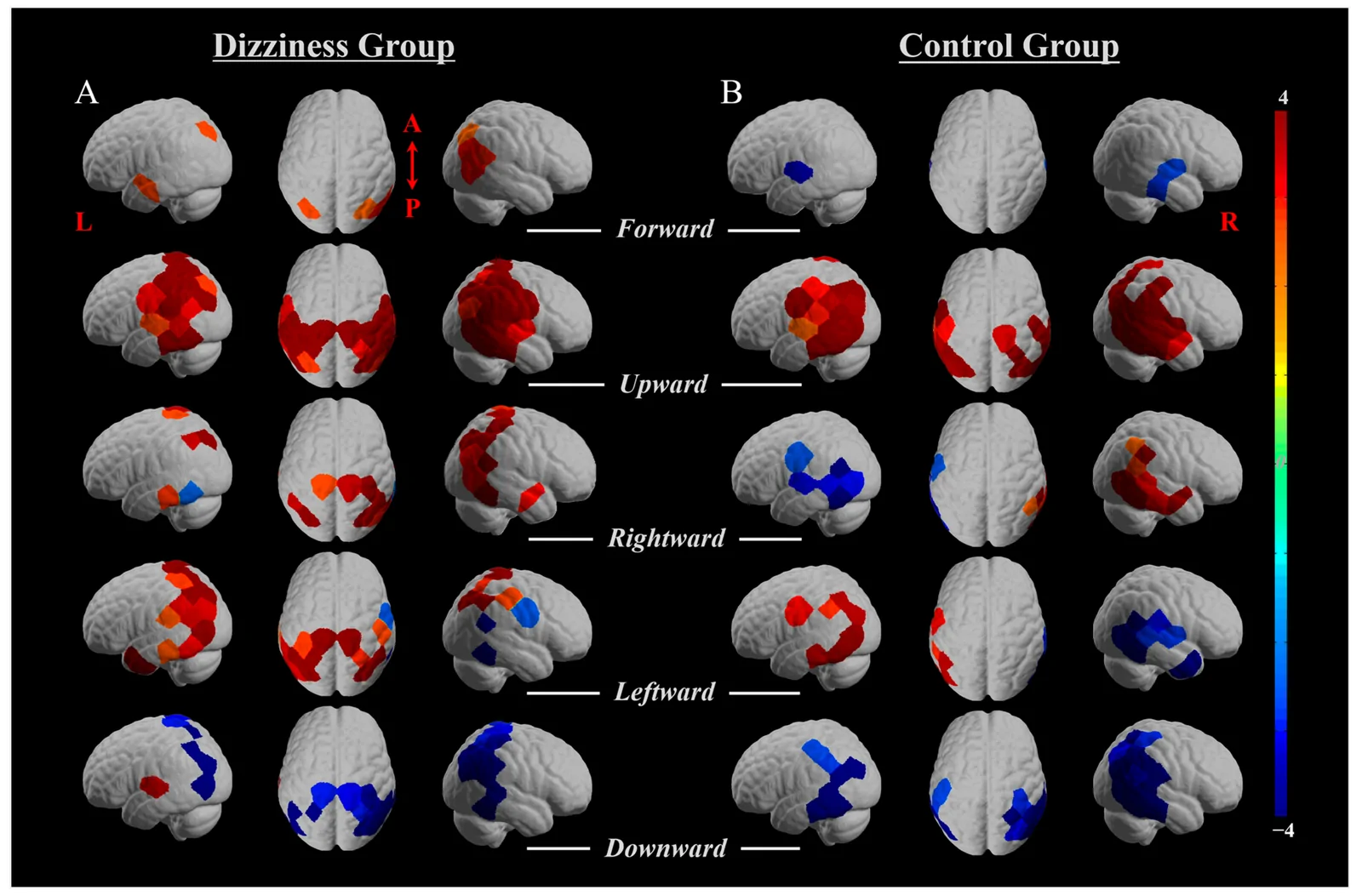
(**A**): t-maps of oxyhemoglobin (HbO) during gait sessions with changes in head posture in the dizziness group. (**B**): t-maps of oxyhemoglobin (HbO) during gait sessions with changes in head posture in the control group. A: anterior side, P: posterior side, L: left hemisphere, R: right hemisphere. These maps are generated by contrasting each session with respect to the baseline (uncorrected *p* < 0.05).

During the forward-looking gait session (FGs), the HbO results showed that the bilateral SPL, MTG, and right AG were significantly activated with respect to the baseline. In the up-looking gait session (UGs), the response results showed significant activation in the bilateral SPL, IPL, STG, MTG, SMG, and AG compared to the baseline. During the right-looking gait session (RGs), the response results showed significant deactivation in the left MTG and significant activation in the bilateral SPL, MTG, the right IPL, the left side of the AG and STG, with respect to the baseline. In the left-looking gait session (LGs), the results showed significant activation patterns of the bilateral SPL and AG, IPL, and the left side of the SMG, STG, and MTG as well as deactivation of the right side of the SMG and MTG with respect to the baseline. During the down-looking gait session (DGs), the response results showed that the bilateral SPL, AG, and MTG and the right side of the IPL and SMG were deactivated, and the responses of the left MTG were activated with respect to the baseline (*p* < 0.05) (Table 2 and Figure 2A).

### 3.2. Cortical Activations of HbO in the Control Group

During the FGs, the activities of the right STG and bilateral MTG were significantly deactivated with respect to baseline. In the UGs, the bilateral SPL, IPL, SMG, AG, STG, and MTG were significantly activated compared with baseline. In the RGs, the right sides of the SMG, AG, and MTG were significantly activated, and the cortices of the left sides of the SMG, AG, and MTG were deactivated with respect to baseline. In the LGs, activation of the left SMG, AG, and MTG and deactivation in the right STG and MTG were observed with respect to baseline. Additionally, deactivation of the right SPL and STG, bilateral IPL, SMG, AG, and MTG was observed in the DGs with respect to the baseline (*p* < 0.05) (Table 3 and Figure 2B).

### 3.3. Comparison of Cortical Activity Between Group Sessions

**Figure 3:**
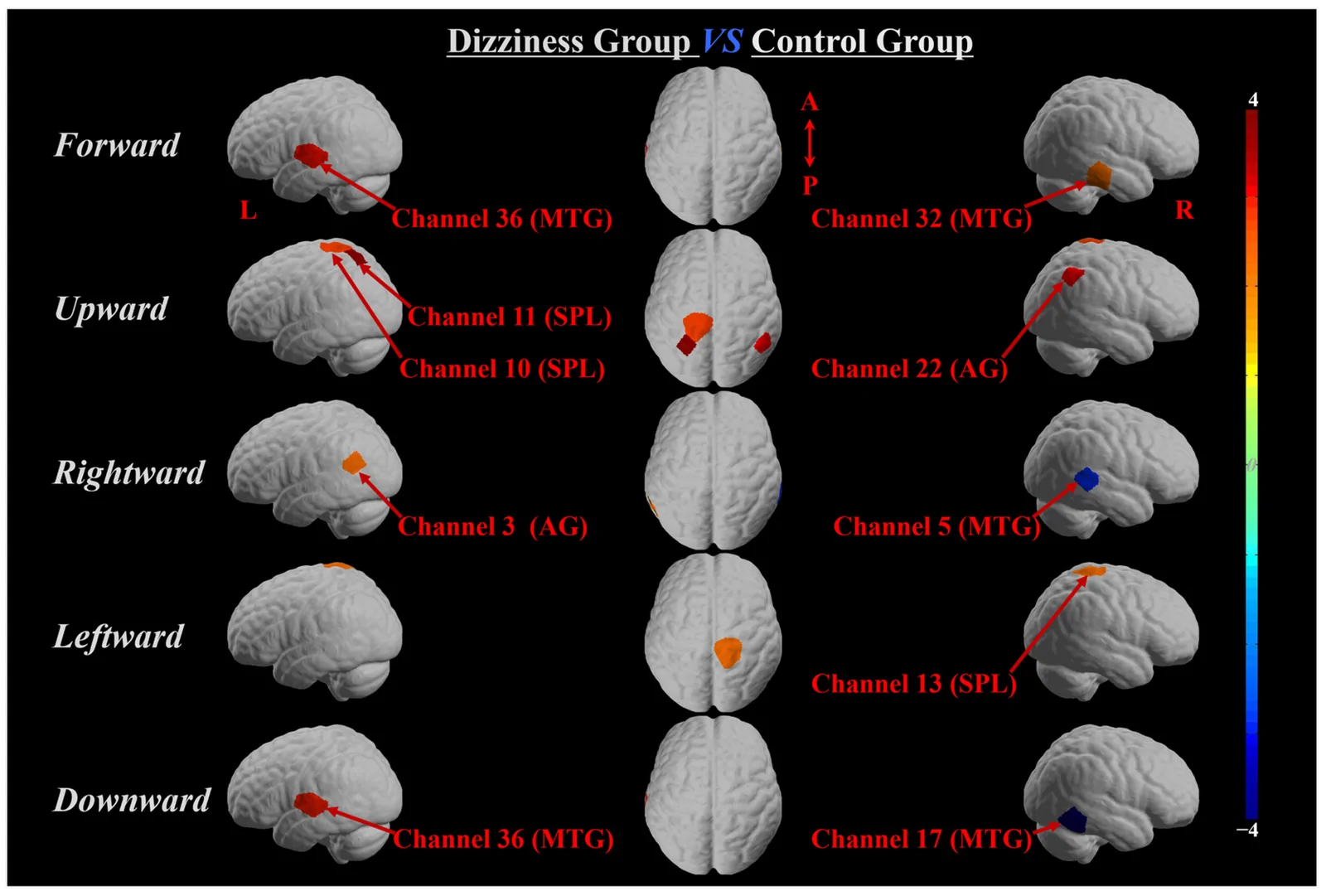
The t-statistic maps of oxyhemoglobin (HbO) corresponding to the contrast between the dizziness group and the control group during gait sessions with changes in head posture. A: anterior side, P: posterior side, L: left hemisphere, R: right hemisphere. These maps are generated by contrasting each session with respect to the baseline (uncorrected *p* < 0.05).

The results revealed a t-statistic map illustrating the HbO response of the experimental groups, showing activation during each experimental session compared to the control group (Table 4 and Figure 3).

The overall results of this study at the uncorrected level (*p* < 0.05) were as follows: (1) During the FGs, it was observed that the results exhibited significant activation in the bilateral MTG. (2) In the UGs, the response results showed that the left SPL and right AG were significantly activated. (3) During the RGs, the dizziness group showed significant activation in the left AG and significant deactivation in the right MTG. (4) In the LGs, significant activation in the right SPL was observed. (5) During the DGs, the response results of the dizziness group showed significant activation of the left MTG and significant deactivation of the right MTG. Notably, while all identified regions exhibited large effect sizes (Cohen’s d > 0.8), only the activation in the left SPL (channel 11) during UGs remained statistically significant after FDR correction (corrected *p* < 0.05).

## 4. Discussion

This study compared cortical activation during gait under different head positions between individuals with and without dizziness, revealing differential responses in visual–vestibular and multisensory integration networks. Specifically, despite similar visual contexts, the asymmetric cortical activities during specific head orientations likely stem from the inherent functional lateralization for spatial processing and the varying cervical proprioceptive inputs driven by distinct neck kinematics [12,13,28,29].

In this study, we found that activity in the left MTG was significantly greater in the dizziness group than in the control group during the FGs and DGs. To maintain balance and reduce sensory conflicts, it is important to ensure the sensory reweighting of the visual and vestibular systems during gait [5]. Specifically, the more reliable the sensory signal, the greater the weight assigned (increased weighting); conversely, the less reliable the sensory signal, the lower the weight assigned (decreased weighting) [30]. Redfern et al. reported that people with imbalance tend to rely on vision to maintain balance [31]. Some neuroimaging studies have investigated the activation of the visual cortex and the deactivation of the vestibular cortex, with results suggesting the importance of the visual–vestibular inhibition and interaction patterns [32]. This pattern reflects the reciprocal inhibitory visual–vestibular interaction and is beneficial for reducing sensory conflicts of the visual–vestibular system [32]. The left MTG (including the motion-sensitive area MT/V5) forms a multisensory integrative network due to its strong interconnection with other multisensory areas and its particular sensitivity to visual motion [12]. The left MTG has also been shown to be sensitive to dizziness [33]. Studies on neural mechanisms have reported decreased volume and greater activation of the left MTG in patients with dizziness than in healthy adults [34,35]. Our results suggest that activation of the left MTG may reflect increased visual weighting in the dizziness group compared with the control group during the FGs and DGs. This may represent a compensatory increase in visual weighting when visual and vestibular signals act simultaneously on posture control [31].

In addition, significantly greater activation of the right MTG was observed during the FGs in the dizziness group compared with the control group. The right MTG is located on the dominant vestibular side in right-handed individuals and is involved in the processing of vestibular inputs and multimodal integration [33]. Previous studies have indicated that deactivation of the vestibular cortex may improve dizziness symptoms in stroke patients [36]. In other words, increased vestibular signals may reflect enhanced cortical processing of vestibular-related information, which is consistent with our results for FGs. As such, greater activation of the right MTG has been associated with increased vestibular inputs, which are reported to be sensitive to dizziness [33].

Furthermore, compared with the control group, the dizziness group exhibited greater deactivation of the right MTG during the RGs and DGs. This pattern may indicate altered vestibular-related cortical processing, which could contribute to sensory conflict during head position changes. Specifically, we also observed greater activation of the left AG during RGs. Some neuroimaging studies have shown the activation of the left AG during visual stimulation and spatial tasks, suggesting that it acts as a cross-modal hub strongly related to the integration of spatial and visual information [37,38]. Additionally, this region has greater functional connectivity with the precuneus, which plays an important role in visual–spatial integration [39]. Based on these studies, we speculate that activation of the left AG might be associated with increased visual weighting. A similar cortical activation pattern was also observed in the control group, which may be consistent with decreased vestibular weighting and increased visual weighting that could contribute to reducing visual–vestibular conflict. This pattern aligns with the sensory reweighting mechanisms described during changes in head posture [13]. Interestingly, in certain clinical populations with specific vestibular deficits, comparable visual–motor strategies, such as looking rightward, may help alleviate dizziness symptoms by reducing sensory conflict [40].

Conversely, during DGs, the deactivation in the right MTG was found in both the dizziness and control groups, with greater deactivation observed in the dizziness group than in the control group. We hypothesize that this specific deactivation pattern may also be linked to altered visual and vestibular sensory weightings. However, in this context, this altered cortical activation might reflect maladaptive changes in vestibular-related cortical processing and sensory integration, potentially contributing to the dizziness symptoms, especially given that looking downward is known to increase diplopia and dizziness [41].

In the present study, the left SPL and right AG were significantly activated in the dizziness group compared to the control group during UGs. While healthy individuals utilize visual cues for postural control when looking upward, individuals with dizziness symptoms rely significantly more on vision to maintain balance and are particularly sensitive to motion under these conditions [31]. As part of the dominant hemisphere of the parietal lobe, the left SPL plays an important role in the transition between the motor and somatosensory cortices, which are involved in the spatial transfer mechanism and integrative function, including somatosensory information from vestibular inputs [42,43]. Some studies on motion sickness have reported that the inhibitory activity of the left SPL is beneficial for reducing the symptoms and onset time of dizziness [44]. Additionally, Morano et al. reported increased activation of the left SPL in patients with dizziness during vertiginous seizures by EEG/fMRI [10]. The right AG is located in the temporoparietal junction and interacts with the parieto-insular vestibular cortex, playing an essential role in the reorienting of spatial attention [45,46]. Therefore, the observed activation of the left SPL and right AG may be consistent with potential altered sensory weighting, which could contribute to sensory conflict and the severity of dizziness. This pattern may specifically reflect the increased sensory demands associated with looking upward during gait.

Additionally, the right SPL was significantly activated in the dizziness group compared with the control group during LGs. The right SPL has been strongly implicated in integrating and processing multisensory inputs [28,47]. The activity of the right SPL is influenced by vestibular inputs [29]. Some structural MRI studies have reported decreased cortical folding of the right SPL in patients with dizziness compared with healthy adults [48]. In particular, an increased BOLD signal in the right SPL in patients with dizziness has been shown in an EEG/fMRI study during vertiginous seizures [10]. Given its role in multisensory integration, we speculate that the greater activation of the right SPL might be related to compensatory changes in vestibular weighting. Based on our results, this specific cortical activation pattern could reflect the underlying neural mechanisms of these sensory reweighting processes, which might ultimately contribute to sensory conflict and increased dizziness intensity.

However, this study had several limitations. First, the relatively small sample size, the restriction to young adults, and the limited ability to evaluate potential sex-related effects may limit the statistical power and generalizability of the findings. Second, subjective dizziness was not assessed after each head position task, preventing direct evaluation of the relationship between cortical activation changes and dizziness severity. Third, participant categorization was based primarily on subjective questionnaires rather than objective neuro-otological examinations, which may have resulted in etiological heterogeneity within the dizziness group and consequently affected the observed cortical activation patterns. Fourth, we did not employ short-separation channels to reduce systemic physiological interference, and the limited penetration depth of fNIRS prevented the assessment of deeper vestibular-related regions, including the cerebellum, insula, and parieto-insular vestibular cortex. Future studies should include larger and more diverse cohorts, incorporate comprehensive objective vestibular assessments, and combine multimodal neuroimaging techniques with improved spatial resolution. Such efforts will help further elucidate the neural mechanisms underlying sensory reweighting during gait and contribute to improving the assessment and rehabilitation of dizziness under different head position conditions.

## 5. Conclusions

These exploratory and hypothesis-generating findings suggest that altered cortical activation associated with multisensory integration may reflect changes in sensory reweighting during gait in individuals with dizziness. The present study provides further insight into the cortical responses associated with dizziness under different head position conditions.

## Figures and Tables

**Table 1:** Comparison of gender, age, DHI and VSS-sf between two groups.

Group	Gender (F/M)	Age	DHI	VSS-sf
Dizziness group (*n* = 8)	8/0	25.00 ± 2.32	27.87 ± 11.01	20.00 ± 4.98
Control group (*n* = 8)	6/2	27.87 ± 4.86	6.62 ± 4.84	2.12 ± 2.79
*p*	0.131	0.234	0.001	<0.001

Mean value ± standard deviation, F: female, M: male, DHI: Dizziness Handicap Inventory, VSS-sf: Vertigo Symptom Scale—short form (*p* < 0.05).

**Table 2:** Significant channels for HbO during each gait condition in dizziness group.

ROIs		Head Posture				
		Forward	Upward	Rightward	Leftward	Downward
Lt	SPL	21	11, 21	10, 21	10, 11, 21	(−)10, 11
	IPL		9, 30		9	
	SMG		2, 31		2	
	AG		3, 19, 20	3, 19	3, 19, 20	(−)3, 19, 20
	STG		4		4	
	MTG	37	1, 15, 35, 36	35, −15	15, 16, 35, 39	36, −16
Rt	SPL	24	13, 14, 24	13, 24	13, 14, 24	(−)13, 14, 24
	IPL		12, 28	12	28	−12
	SMG		6, 29		−29	−6
	AG	23	22, 23	22, 23	22	(−)22, 23
	STG		8, 33			
	MTG	7, 18	5, 7, 17, 18, 32	7, 17, 18, 34	(−)7, 17	(−)5, 7, 17

HbO: oxyhemoglobin, ROIs: regions of interest, Lt: left, Rt: right, SPL: superior parietal lobule, IPL: inferior parietal lobule, SMG: supramarginal gyrus, AG: angular gyrus, STG: superior temporal gyrus, MTG: middle temporal gyrus, −/(−): deactivation channel.

**Table 3:** Significant channels for HbO during each gait condition in control group.

ROIs		Head Posture				
		Forward	Upward	Rightward	Leftward	Downward
Lt	SPL		21			
	IPL		30			−30
	SMG		2, 31	−31	2, 31	−2
	AG		3, 19, 20	−3	19, 20	(−)3, 20
	STG		4			
	MTG	−36	1, 15, 16, 35, 36	(−)1, 15, 16, 36	15, 16, 35	(−)1, 15, 35, 36
Rt	SPL		13, 14, 24			(−)14, 24
	IPL		28			−12
	SMG		6	6		−6
	AG		23	22		(−)22, 23
	STG	−33	8, 33		(−)8, 33	−8
	MTG	−32	5, 7, 17, 18, 32, 34	5, 7, 17, 18, 32, 34	(−)5, 7, 17, 18, 38	(−)5, 7, 17, 18, 32

HbO: oxyhemoglobin, ROIs: regions of interest, Lt: left, Rt: right, SPL: superior parietal lobule, IPL: inferior parietal lobule, SMG: supramarginal gyrus, AG: angular gyrus, STG: superior temporal gyrus, MTG: middle temporal gyrus, −/(−): deactivation channel.

**Table 4:** Comparison of HbO response between two groups during gait sessions.

Head Posture	ROIs	Channel	t Value	*p_uncorrected_*	*p_corrected_*	Cohen’s d
Forward	Lt. MTG	36	2.92	0.011 *	0.218	1.46
	Rt. MTG	32	2.17	0.047 *	0.616	1.09
Upward	Lt. SPL	10, 11	2.58/4.28	0.022 */<0.001 ***	0.284/0.031 *	1.29/2.14
	Rt. AG	22	3.05	0.009 **	0.169	1.53
Rightward	Lt. AG	3	2.15	0.025 *	0.644	1.08
	Rt. MTG	5	−2.72	0.017 *	0.325	−1.36
Leftward	Rt. SPL	13	2.20	0.045 *	0.450	1.10
Downward	Lt. MTG	36	2.81	0.014 *	0.277	1.41
	Rt. MTG	17	−3.88	0.002 **	0.066	−1.94

HbO: oxyhemoglobin, ROIs: regions of interest, Lt: left, Rt: right, MTG: middle temporal gyrus, SPL: superior parietal lobule, AG: angular gyrus. * *p* < 0.05, ** *p* < 0.01, *** *p* < 0.001.

## Data Availability

The data presented in this study are available from the corresponding author upon reasonable request. The data are not publicly available due to privacy and ethical restrictions involving human participants.

## Abbreviations

The following abbreviations are used in this manuscript:

fNIRS	Functional Near-Infrared Spectroscopy
HbO	Oxygenated Hemoglobin
SPL	Superior Parietal Lobule
IPL	Inferior Parietal Lobule
SMG	Supramarginal Gyrus
AG	Angular Gyrus
STG	Superior Temporal Gyrus
MTG	Middle Temporal Gyrus
